# The number of metastatic lymph nodes optimizes staging in patients aged 55 years or older with papillary thyroid cancer

**DOI:** 10.3389/fendo.2022.1026737

**Published:** 2022-12-09

**Authors:** Yun-Gang Sun, Fei Chen, Qiao-Ling Sun, Jin-Yu Tian, Xiao-Chuan He

**Affiliations:** ^1^ Department of Nuclear Medicine, Zhujiang Hospital, Southern Medical University, Guangzhou, China; ^2^ Department of Thyroid Surgery, Zhujiang Hospital, Southern Medical University, Guangzhou, China

**Keywords:** papillary thyroid cancer, number of positive lymph nodes, TNM staging system, prognosis, SEER

## Abstract

**Purpose:**

Current staging criteria for papillary thyroid cancer (PTC) do not include the number of metastatic lymph nodes (LNs), which is highly predictive of survival in multiple cancers. The LN metastasis burden is particularly relevant for older adults with thyroid cancer because of their poor prognosis. We examined a modified staging system for this population utilizing node number (Nn).

**Methods:**

Overall, 14,341 patients aged 55 years or older with stage I-IVB PTC were identified in the 2004–2015 Surveillance, Epidemiology and End Results database. Cox regression models were conducted to test the relationship between positive LN number and PTC-specific survival (PTCSS). Independent training/validation sets were used to derive and validate a new revised TNnM grouping. The 8th edition American Joint Committee on Cancer TNM staging system was compared with TNnM stage by calculating the 10-year PTCSS rates, Harrell’s concordance index (C-index), and Akaike’s information criterion (AIC).

**Results:**

An increase in number of LN metastases was identified as an independent, negative prognostic factor for PTCSS in multivariate analysis. 10-year PTCSS for stage I-IVB based on the AJCC 8th edition TNM were 98.83%, 93.49%, 71.21%, 72.95%, and 58.52%, respectively, while 10-year PTCSS for the corresponding stage in the TNnM were 98.59%, 92.2%, 83.26%, 75.24%, and 56.73%, respectively. The revised TNnM stage was superior, with a higher C-index and a lower AIC in both the training and validation cohorts.

**Conclusion:**

The TNnM staging system for PTC patients ≥ 55 years could be associated with improved outcomes. External validation studies of this system are warranted.

## Introduction

Papillary thyroid cancer (PTC) is the most common endocrine malignancy, and its incidence has risen rapidly worldwide over the last 30 years (1, 2). In the United States, in 2021, approximately 42% of patients who were newly diagnosed with thyroid cancer were ≥ 55 years of age (3), and this proportion is projected to grow as the population ages (4). Older patients with PTC present more often with more advanced diseases, larger median tumor sizes, higher rates of node positivity, and higher propensities for distant metastatic spread when compared to their younger counterparts (5–7). In addition, the older age group accounts for the overwhelming majority of thyroid cancer deaths (6–8). Given the poor overall prognosis of PTC in older adults, there is a strong need for more precise staging methods to predict patient survival and help tailor individualized treatment approaches.

According to the 8th edition of the American Joint Committee on Cancer (AJCC) staging system for PTC, among patients who are diagnosed at 55 years of age or older, positive nodal involvement in the absence of gross extrathyroidal extension or distant metastases is considered as stage II disease. However, this stage group classification considers only one lymph node (LN) factor: N0 (no positive nodes) or NX (regional nodes cannot be assessed) versus an N1 (positive nodes) status, and it does not account for the number of metastatic LNs (9), which is a well-established predictor of mortality and included in the AJCC staging for a variety of other cancers (10–15). Prior studies have proposed modifications to the definitions of the AJCC (8th edition) staging manual for PTC. The results from these studies suggest that incorporation of the following additional prognostic factors into the AJCC 8th edition can create prognostically accurate cancer staging systems: multiple age cutoffs, American Thyroid Association risk stratification system, and comorbidities (16–18). However, to date, there is a paucity of data on the prognostic performance of proposed stage schemes by the inclusion of involved node number. Moreover, while a previous study has demonstrated the prognostic significance of positive LN numbers in younger patients with PTC (19), few studies have examined the impact of the number of nodal metastases in older adults. Thus, whether the positive node number is optimal for PTC prognosis of this population or whether replacement of the current nodal classification (e.g., N0, N1a, N1b) with LN number would result in better overall prognostic stage groupings remains unknown.

The number of metastatic LNs detected is dependent on the extent of LN dissection and the intensity of pathologic evaluation. In this condition, in addition to the AJCC N stage, some studies have suggested that LN status should be described by LN density (LND) (20, 21), which is the number of positive LNs divided by the total number of harvested nodes. LND correlates with the total LN yield and is a surrogate of adequacy of neck dissection. Several articles have shown that use of the LND allowed investigators to better stratify the prognosis after resection of malignant neoplasms of the head and neck (22, 23). However, debate exists about whether the most accurate prognostic factor in PTC patients is the number of positive LNs or the LND.

In this study, given that the relative importance of other LN factors, such as number and LND, has not been systemically investigated previously in older patients with PTC, we use population-based data to evaluate the association of the numerical metastatic nodal disease burden with PTC-specific survival (PTCSS) among patients with PTC who were aged ≥ 55 years at the time of diagnosis and propose modified N category-based AJCC TNM staging systems that may provide patients with more accurate survival estimates.

## Materials and methods

### Study population

**Figure 1:**
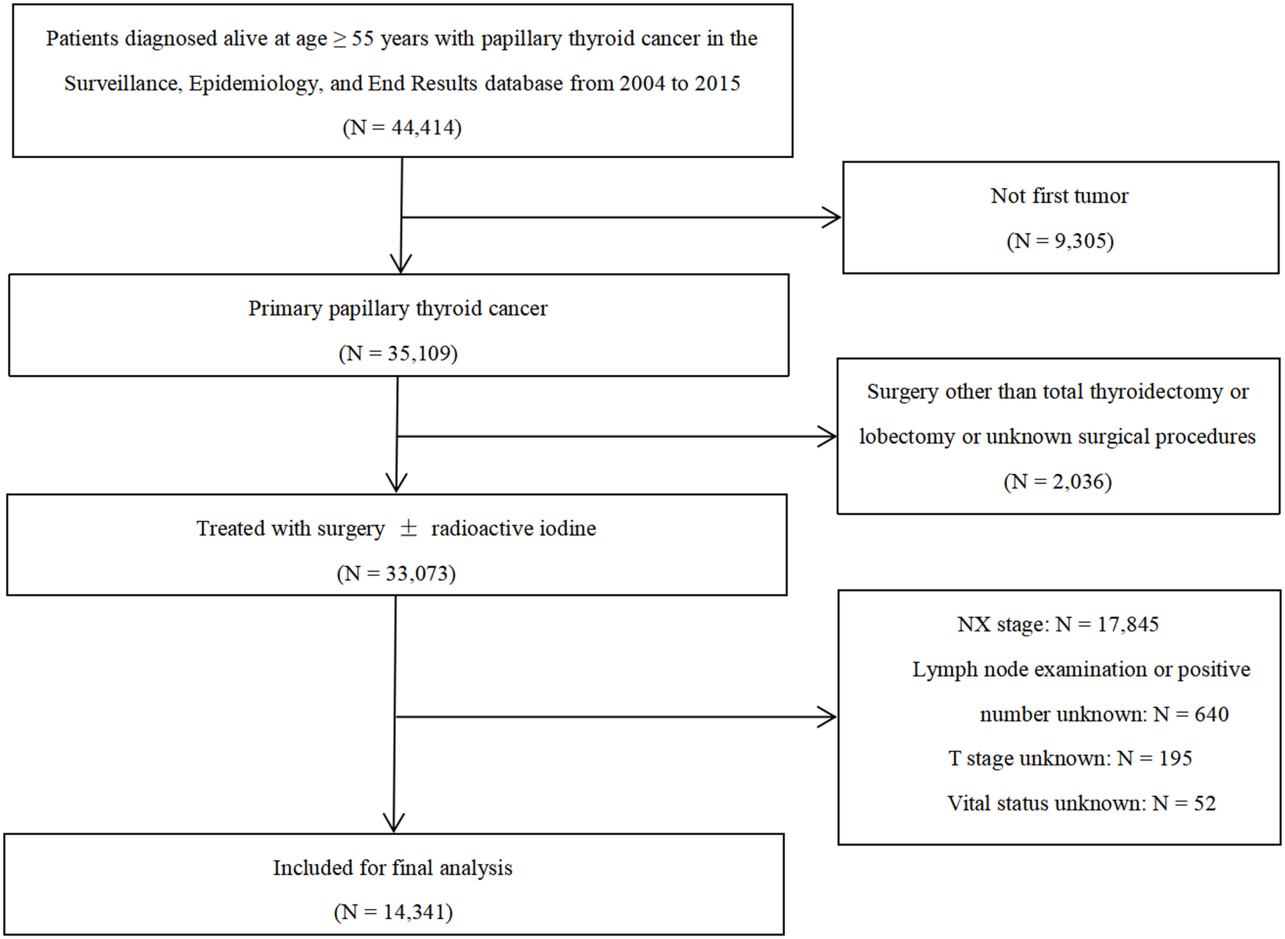
Flow chart of the study cohort.

We conducted a retrospective cohort study using the Surveillance, Epidemiology, and End Results (SEER) database, which collects data on cancer occurrences in 18 geographic regions covering 28% of the US population (24). Individuals who were aged ≥ 55 years with a diagnosis of PTC (International Classification of Diseases for Oncology, third edition histology codes 8050/3, 8260/3, and 8340-8344/3), between 2004 and 2015, were assessed (n = 44,414). 9,305 patients were excluded due to previous cancers. Patients not undergoing surgery of a primary site, removal of less than a thyroid lobe, or with unknown surgery status were eliminated (n = 2,036). Patients who had no node examined (n = 17,845), incomplete information on stage of disease (n = 195) or LN data (n = 640), or missing vital status (n = 52) were also excluded, leaving 14,341 eligible individuals for the study (**Figure 1**). To establish the prognostic model, the cohort was split into a training set containing the sample diagnosed with PTC between 2004 and 2009, and a validation set including the participants diagnosed between 2010 and 2015. The research protocol used de-identified data and was exempted by the Southern Medical University from institutional review board review.

Variables obtained within the SEER database included patient age at the time of diagnosis, sex (male or female), race (white, black, other, or unknown), period of diagnosis, tumor size, tumor extension, nodal stage, presence of distant metastases (yes or no), number of nodes removed, number of nodal metastases, extent of thyroidectomy (thyroid lobectomy or total thyroidectomy), RAI therapy (yes or no), survival months, and cause of death. The LN density (LND) was defined as the ratio of the number of positive nodes to the number of nodes removed. Lobectomy was defined as removal of a thyroid lobe with or without isthmectomy, and total thyroidectomy included total, near-total, or subtotal thyroid resection. RAI was documented as if patients received radioisotopes. Stages were classified based on the guidelines of the AJCC 8th edition. PTCSS was defined as the time interval (in months) from surgery until death from PTC.

### Statistical analysis

We summarized baseline patient data using descriptive statistics. We estimated survival curves *via* the Kaplan–Meier method, and we used log-rank tests to compare differences in survival. For correlation of prognostic factors with survival in PTC, we carried out univariate and multivariable analyses by employing Cox regression models. Prognostic factors were kept in the multivariate model only if statistically significant on univariable analysis. Using X-tile software 3.6.1 version *via* an internal cross-validation method based on the minimum *P* values from log-rank χ^2^ statistics (Rimm Lab, New Haven, CT) (25), we identified the optimal cut-off points for positive node number and defined a new Nn category. To propose revised TNnM stages, first, we divided the entire training cohort into 16 data subsets using varying T, Nn, and M category combinations (eTable in the supplement). Then, these subsets were amalgamated into 5 homogeneous categories consistent with those of the current TNM stage groupings, based on the principle of similar 10-year PTCSS estimate within the same stage grouping and maximum differences in 10-year PTCSS estimate among different stage groupings. A concordance index (C-index) was computed to compare the discrimination ability of the proposed TNnM and AJCC 8th edition system (26). Internal validation using standard bootstrapping techniques (1000 replications) was performed, and optimism-corrected estimates of the C-index were calculated for each system. Akaike’s information criterion (AIC) was also reported for each model. The AIC provided a relative measure of model quality; smaller values correspond with a better fitting model. As a general guideline, differences ≥ 10 indicate substantial improvement in the fit of the model (27). Significance between the C-index of the revised TNnM and current TNM stage models was determined using the Z testing method in the “CsChange” package in R (28). Analyses were conducted using R, version 4.0.3 (R Foundation). Two-sided *P* value < 0.05 was considered statistically significant.

## Results

### Patient cohort

Between January 2004 and December 2015, a total of 14,341 patients who were aged 55 years or older with stage I to IVB PTC met inclusion criteria (**Figure 1**), thereof 4,971 patients in the training set and 9,370 patients in the validation set. Patient baseline characteristics for the two groups are displayed in **Table 1**. The nodal staging of the entire study cohort was N0, N1a, N1b, and unknown was 62.5%, 20.9%, 14.4%, and 3.4%, respectively. The mean number of positive LNs, LN count, and LND was 1.6, 6.5, and 0.2, respectively, for the training cohort and 1.8, 8.0, and 0.2, respectively, for the validation cohort.

**Table 1:** Patient and tumor characteristics.

Characteristic	Total, n (%) or Mean (SD)	Training Set, n (%) or Mean (SD)	Validation Set, n (%) or Mean (SD)
Total, n (%)	14,341 (100)	4,971 (34.7)	9,370 (65.3)
Age, mean (SD), years	64.0 (7.5)	64.1 (7.7)	63.9 (7.3)
Sex			
Female	10,277 (71.7)	3,558 (71.6)	6,719 (71.7)
Male	4,064 (28.3)	1,413 (28.4)	2,651 (28.3)
Race			
White	12,046 (84.0)	4,210 (84.7)	7,836 (83.6)
Black	551 (3.8)	178 (3.6)	373 (4.0)
Other/unknown	1,744 (12.2)	583 (11.7)	1,161 (12.4)
T category			
T1	9,326 (65.0)	3,107 (62.5)	6,219 (66.4)
T2	2,896 (20.2)	982 (19.8)	1,914 (20.4)
T3	1,003 (7.0)	341 (6.9)	662 (7.1)
T4a	794 (5.5)	374 (7.5)	420 (4.5)
T4b	322 (2.2)	167 (3.4)	155 (1.7)
N category			
N0	8,963 (62.5)	3,042 (61.2)	5,921 (63.2)
N1a	2,893 (20.9)	988 (21.0)	1,905 (20.8)
N1b	1,996 (14.4)	671 (14.3)	1,325 (14.5)
Unknown	489 (3.4)	270 (5.4)	219 (2.3)
M category			
M0	13,687 (95.4)	4,694 (94.4)	8,993 (96.0)
M1	654 (4.6)	277 (5.6)	377 (4.0)
Overall TNM stage			
I	8,190 (57.1)	2,733 (55.0)	5,457 (58.2)
II	4,623 (32.2)	1,526 (30.7)	3,097 (33.1)
III	669 (4.7)	321 (6.5)	348 (3.7)
IVA	205 (1.4)	114 (2.3)	91 (1.0)
IVB	654 (4.6)	277 (5.6)	377 (4.0)
No. of metastatic LNs, mean (SD)	1.7 (4.1)	1.6 (3.7)	1.8 (4.3)
No. of LNs examined, mean (SD)	7.5 (12.2)	6.5 (10.8)	8.0 (12.8)
LND, mean (SD)	0.2 (0.3)	0.2 (0.3)	0.2 (0.3)
Extent of surgery			
Thyroid lobectomy	992 (6.9)	375 (7.5)	617 (6.6)
Total thyroidectomy	13,349 (93.1)	4,596 (92.5)	8,753 (93.4)
RAI administration			
No	6,918 (48.2)	2,208 (44.4)	4,710 (50.3)
Yes	7,423 (51.8)	2,763 (55.6)	4,660 (49.7)

SD, standard deviation; RAI, radioactive iodine; LN, lymph node; LND, lymph node density.

### Prognostic impact of the number of metastatic lymph nodes

During a median follow-up of 125 months (range, 0 to 179 months), a total of 372 patients (7.5%) aged 55 years or older died of thyroid cancer in the training cohort. Factors correlated with PTCSS are listed in **Table 2**. In the univariate analysis, an increasing number of metastatic nodes was associated with significantly compromised PTCSS, with a hazard ratio (HR) of 1.09 (95% confidence interval [CI] 1.08–1.11; *P* < 0.001). On multivariable analysis, increasing involved node number remained a significant independent predictor of poor PTCSS (HR 1.02, 95% CI 1.00–1.04; *P* = 0.041). Additionally, the results also revealed that both stage N1a and N1b were independent risk factors for survival (both *P* < 0.001). However, LND had no independent impact on survival (*P* = 0.8).

**Table 2:** Prognostic factors associated with cancer-specific mortality.

	Univariable Analysis		Multivariable Analysis	
Characteristic	HR (95% CI)	*P* value	HR (95% CI)	*P* value
No. of metastatic LNs	1.09 (1.08–1.11)	<0.001	1.02 (1.00–1.04)	0.041
Age, years	1.09 (1.07–1.10)	<0.001	1.06 (1.05–1.07)	<0.001
Sex				
Mmale	1 (Reference)		1 (Reference)	
Female	0.44 (0.36–0.54)	<0.001	0.82 (0.66–1.01)	0.063
Race				
White	1 (Reference)		1 (Reference)	
Black	0.47 (0.21–1.04)	0.06	0.50 (0.22–1.12)	0.092
Other/unknown	1.44 (1.09–1.91)	0.01	0.93 (0.70–1.23)	0.612
T category				
T1	1 (Reference)		1 (Reference)	
T2	4.99 (3.49–7.14)	<0.001	3.46 (2.40–4.97)	<0.001
T3	12.56 (8.64– 18.26)	<0.001	7.43 (5.06–10.91)	<0.001
T4a	25.87 (18.58–36.03)	<0.001	11.77 (8.30–16.70)	<0.001
T4b	32.44 (22.37–47.05)	<0.001	12.83 (8.64–19.05)	<0.001
N category				
N0	1 (Reference)		1 (Reference)	
N1a	4.32 (3.23–5.80)	<0.001	2.25 (1.54–3.30)	<0.001
N1b	9.99 (7.60–13.12)	<0.001	2.63 (1.79–3.87)	<0.001
Unknown	5.82 (3.97–8.53)	<0.001	2.08 (1.32–3.27)	<0.001
M category				
M0	1 (Reference)		1 (Reference)	
M1	9.85 (7.86–12.33)	<0.001	2.92 (2.27–3.77)	<0.001
LND	4.63 (3.65–5.86)	<0.001	0.95 (0.67–1.37)	0.8
Extent of surgery				
Thyroid lobectomy	1 (Reference)		1 (Reference)	
Total thyroidectomy	2.50 (1.41–4.44)	0.002	1.20 (0.67–2.15)	0.53
RAI administration				
No	1 (Reference)		‐	‐
Yes	1.22 (0.99–1.50)	0.067	‐	‐

HR, hazards ratio; CI, Confidence interval; LN, lymph node; RAI, radioactive iodine; LND, lymph node density.

### Derivation and performance of the TNnM stage groups

**Figure 2:**
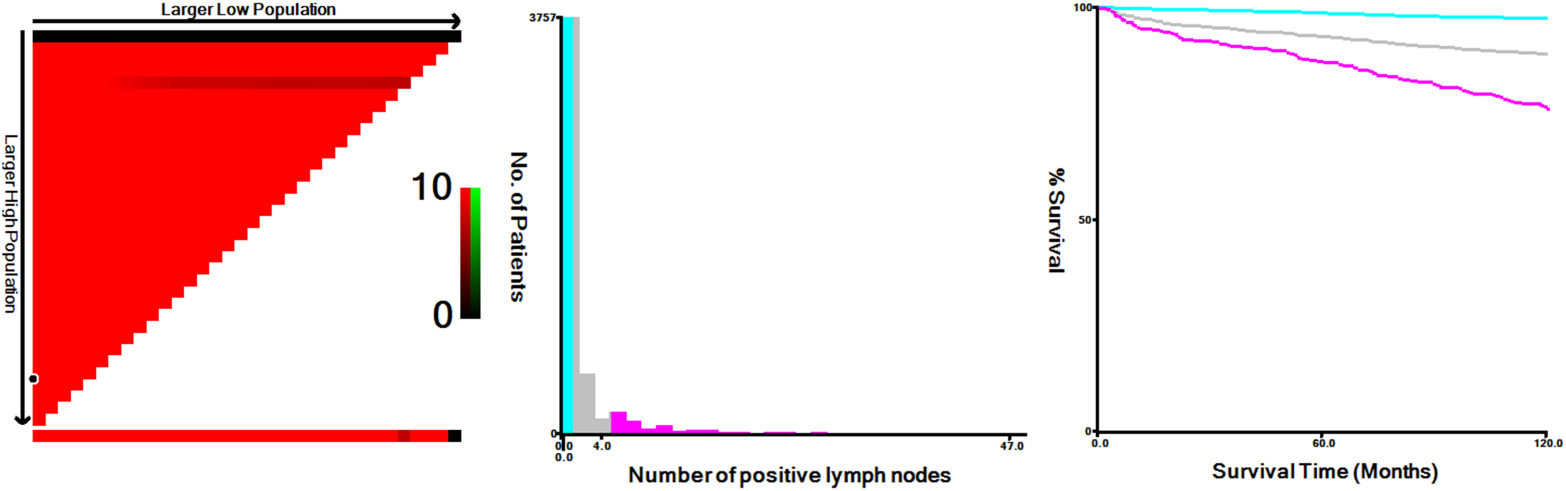
X-tile analysis of 10-year disease-specific survival using patients’ data in SEER registry. The optimal cut-off values for the positive node number were 0, 1 and 4 in the training cohort (χ^2^ = 333.0712, *P* < 0.0001).

Using X-tile, we have identified optimal cut-off points of the positive node number, which were 0, 1, and 4 in the training set (**Figure 2**). Based on this result, we proposed a revised Nn staging criteria, in which N0n indicates no evidence of LN metastasis, N1n indicates 1 to 4 metastatic nodes, and N2n indicates > 4 metastatic nodes. These patients in the training cohort were classified into 16 TNnM groups corresponding to different combinations of 8th edition AJCC T/M and Nn classification (1): T1N0nM0 (2), T2N0nM0 (3), T3N0nM0 (4), T4aN0nM0 (5), T4bN0nM0 (6), T1N1nM0 (7), T2N1nM0 (8), T3N1nM0 (9), T4aN1nM0 (10), T4bN1nM0 (11), T1N2nM0 (12), T2N2nM0 (13), T3N2nM0 (14), T4aN2nM0 (15), T4bN2nM0, and (16) T-anyNn-anyM1 (eTable in the supplement). Review of the 10-year PTCSS estimates for the above 16 groups showed that the patients could be amalgamated into the following proposed stage groupings: stage I (T1-2N0nM0 or T1N1nM0); stage II (T3N0nM0 or T2N1nM0 or T1-2N2nM0); stage III (T4N0nM0 or T3N1nM0); stage IVA (T4N1nM0 or T3N2nM0); stage IVB (T4N2nM0 or T-anyNn-anyM1) (**Table 3**). Under the proposed TNnM groupings of the 4,971 patients, 3,388 (68.2%) were stage I, 719 (14.5%) stage II, 215 (4.3%) stage III, 265 (5.3%) stage IVA, and 384 (7.7%) stage IVB (**Table 3**). The adjusted multivariable survival analysis showed a significant trend for an increased risk of PTC-specific mortality among older patients in each successive TNnM stage grouping (*P* for trend < 0.001).

**Table 3:** Survival for AJCC 8th edition TNM and TNnM staging systems for patients aged ≥ 55 years with papillary thyroid cancer.

Classification System	Criteria	No. (%) of Patients	10-year PTCSS, %	HR (95% CI)^a^
AJCC 8th edition TNM stage				
I	T1-2N0M0	2,733 (55)	98.83	1 (Reference)
II	T1-2N1M0 T3, any N, M0	1,526 (30.7)	93.49	5.85 (3.81–9.00)
III	T4a, any N, M0	321 (6.5)	71.21	22.30 (14.41–34.49)
IVA	T4b, any N, M0	114 (2.3)	72.95	24.60 (14.73–41.05)
IVB	Any T, any N, M1	277 (5.6)	58.52	37.78 (24.50–58.26)
TNnM stage				
I	T1-2N0nM0 or T1N1nM0	3,388 (68.2)	98.59	1 (Reference)
II	T3N0nM0 or T2N1nM0 or T1-2N2nM0	719 (14.5)	92.2	5.95 (4.01–8.86)
III	T4N0nM0 or T3N1nM0	215 (4.3)	83.26	11.22 (7.22–17.44)
IVA	T4N1nM0 or T3N2nM0	265 (5.3)	75.24	17.67 (11.73–26.60)
IVB	T4N2nM0 or any T, any Nn, M1	384 (7.7)	56.73	33.45 (23.42–47.78)

PTCSS, papillary thyroid cancer-specific survival; Nn, metastatic node number; N0n, no evidence of metastatic nodes; N1n, 1-4 metastatic nodes; N2n, > 4 metastatic nodes; AJCC, American Joint Committee on Cancer; HR, hazards ratio; CI, confidence interval.

a HRs were generated from 2 different multivariable Cox proportional hazards models based on the proposed TNnM and current TNM staging system. Both models were adjusted for patient age, sex, race, surgery, radioactive iodine use, and lymph node density.

**Figure 3:**
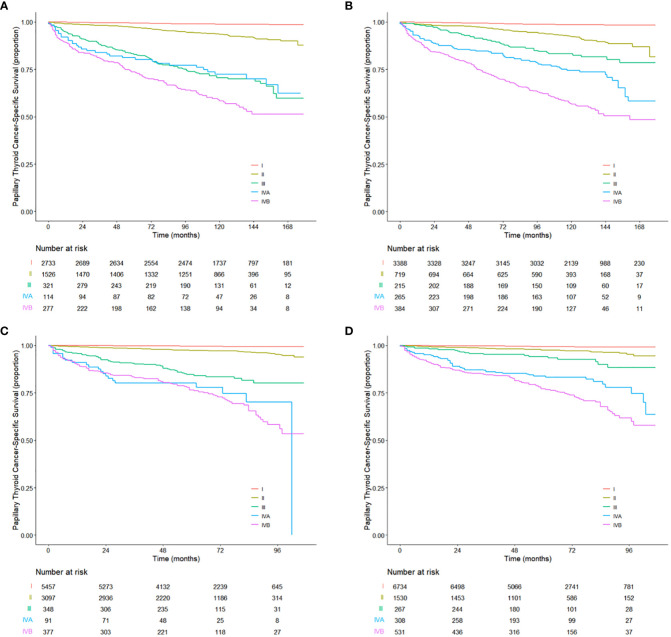
Kaplan-Meier estimates for the patients aged 55 years or older from **(A)** the training cohort and **(C)** the validation cohort using the current TNM staging system. Kaplan-Meier estimates for the patients aged 55 years or older from **(B)** the training cohort and **(D)** the validation cohort using the revised TNnM staging system.

Kaplan–Meier survival graphs for the TNnM and current TNM staging are illustrated in **Figure 3**. The 10-year PTCSS rates for I-IVB AJCC stages were 98.83, 93.49, 71.21, 72.95, and 58.52%, respectively (log-rank *P* < 0.001, **Figure 3A**), while that for I-IVB stages of the proposed TNnM groupings were 98.59, 92.2, 83.26, 75.24, and 56.73%, respectively (log-rank *P* < 0.001, **Figure 3B**). Of note, no significant difference was observed in the survival rate between the current AJCC stage III and IVA disease (log-rank *P* = 0.86). In contrast, the revised TNnM system demonstrated an improvement in separation of stage III versus IVA disease (log-rank *P* = 0.008).

Furthermore, the revised TNnM stage grouping had better PTCSS predictive ability than the AJCC 8th edition system (indicated by the higher C-index: 0.871, 95% CI 0.852–0.889 and 0.855, 95% CI 0.836–0.873, for the models with the TNnM and AJCC, respectively; difference in C-index 0.016, 95% CI 0.007–0.026, *P* < 0.001). The optimism-corrected, bootstrapped C-index values were similar after the internal validation (0.869 vs. 0.853). The revised TNnM system also provided a better fit for the data (indicated by the lower AIC value: 5347 vs. 5458).

### Validation in an independent cohort

The utility of the revised TNnM staging system was subsequently evaluated in an independent validation cohort of 9,370 patients aged ≥ 55 years with PTC from the 2010–2015 SEER database, using the same analyses as for the training cohort. The median follow-up period for the validation data set was 64 months (range, 0 to 107 months). There were 288 death (3.1%) events attributed to PTC. Notably, when stratifying patients by the 8th edition AJCC system, no significant difference in PTCSS was seen in patients with stage IVA disease compared with those with stage IVB diseases (HR 1.10, 95% CI 0.69–1.76; *P* = 0.7; **Figure 3C**). However, the survival curves between stage IVA and IVB disease in the revised TNnM system exhibited were clearly distinguishable (HR 1.52, 95% CI 1.10–2.09; *P* = 0.01; **Figure 3D**). The proposed TNnM system outperformed the current AJCC TNM system in the validation population, with a higher C-index in discrimination (0.900 vs. 0.886; difference in C-index 0.014, 95% CI 0.03–0.025, *P* = 0.015) and better statistical model fit with lower AIC (4263 vs. 4339).

## Discussion

An accurate staging system is critical to convey the extent of disease, help guide treatment selection, and inform prognosis. Currently, the 8th edition TNM classification of differentiated thyroid cancer proposed by the AJCC is being followed in clinical practice. This system for PTC incorporates LN positive and the anatomic location of positive LNs (9), but does not consider the total number of tumor-positive nodes as a staging variable, which is characterized as a predominant prognostic factor in most head and neck cancer patients (14, 19, 29). Thus, it is necessary to develop alternative stage groupings including positive node number that could be used to further refine prognostic information.

In this large study of 14,341 patients 55 years or older who underwent surgery for PTC, we showed an increasing number of LN metastases to be significantly associated with compromised PTCSS. Furthermore, we re-defined the regional LN classification according to the total number of metastatic positive nodes: N0n, no node metastasis; N1n, 1–4 metastatic nodes, and N2n, > 4 metastatic nodes. We further used objective criteria to create a new, internally validated TNnM staging with performance superior to that of the traditional 8th edition TNM staging. To our knowledge, no modified disease-specific staging schemes that incorporate the number of metastatic nodes exist for older patients with AJCC stage I to IVB PTC. Once externally validated in large cohorts, this system could then have the potential to become an evidence-based adjunct aiding in personalized treatment, discussions of prognosis, and stratification of future clinical trials for PTC patients aged 55 years or older.

Our findings support a previous study by Adam et al. (19), who showed that in a cohort of 30,193 individuals with PTC, the number of metastatic cervical LNs is a critical predictor of overall mortality. However, the study by Adam et al. included only patients who were diagnosed at < 45 years of age without distant metastases, and their study was conducted over an earlier and longer period than our own (1988–2006 vs. 2004–2015). Similar risk estimates between increasing positive node numbers and compromised survival have also been observed in two recent studies (30, 31). The results of our study add to these findings by its adjustment for important LN parameters, such as nodal stage and LNR, and by focusing exclusively on patients who were aged ≥ 55 years, at a relatively high risk of mortality from thyroid cancer, and accounted for a growing proportion of patients with PTC with increasing life expectancy. Our work suggests that the number of LNs involved would have a substantial proposed revision for the current AJCC staging system for the older population.

The TNnM stage is consistent with previous studies which have demonstrated the significance of cervical LN features in prognosis for patients with PTC. In a retrospective analysis of 2,542 patients with PTC from the MD Anderson between 2000 and 2015 (21), a new modified TNM staging schema was proposed, incorporating LN ratio in the current AJCC system, which stratified the risk groups better with respect to overall survival and PTCSS than the traditional categorical TNM system. A limitation of that analysis was that it did not adjust for the total number of nodal metastases, which may, at least partly, affect the relationship between LN ratio and patient survival. In addition, this study did not report in detail how to derive the LN ratio-based staging system, but only listed the final stage groupings. A recent study from the Republic of Korea that included 745 N1b PTC patients without distant metastases found an increase in predictive ability (32), with a C-index of 0.84 and 0.87 for the 8th edition TNM classification and the alternative prognostic grouping using lateral LN ratio and largest LN size, respectively. The result that positive LN number rather than LN ratio was statistically associated with survival in our series is in disagreement with the above two studies. One possible explanation could be variances in the population chosen, the therapeutic standards used, the duration of the follow-ups, and the methodologies and statistics considered.

Existing staging guidelines for PTC remove nodal factors (e.g., location, size, or number of nodes involved) from stage III or IV disease; such revisions may not be regarded as clinically appropriate for older patients. Indeed, the AJCC 8th edition system failed to clearly distinguish PTCSS rates of the patients between stages III and IVA in this study. Similar to our findings, Tam et al. performed a retrospective review of 2,579 patients with differentiated thyroid cancer (33), 56 had AJCC 8th edition stage III tumors, and 67 had stage IV tumors. They did not find any statistically significant differences in disease-specific survival between stage III and IV patients. Using a new revised N classification according to the positive node number, the proposed TNnM staging system could resolve all overlaps of survival estimates in the AJCC stages in both the derivation and validation data sets. In addition, C-index and AIC confirm the predictive superiority of this proposed TNnM system over the current 8th edition AJCC TNM system.

Recently, several cohort studies on the Asian population have also tried to modify the eighth edition of the TNM staging system for PTC by incorporating a variety of clinicopathologic characteristics of metastatic LNs. Li et al. and colleagues (34) conducted a single-center retrospective study, including 6,165 Chinese individuals, which found that PTC patients with extranodal extension present worse PTCSS and incorporating extranodal extension in TNM classification identifies poor-risk patients more accurately. Moreover, two studies have demonstrated that sub-classification based on the location of the metastatic LNs in older patients with stage I/II can predict disease-specific mortality more precisely than the current AJCC TNM system in the Korean population (35, 36). In a study involving Japanese patients (37), investigators revealed that the revised TNM staging employing the size of positive nodes could be more useful for the prediction of PTCSS. However, until now, few data have been available on variation in the prognostic implication of nodal metastases, as well as that of concurrent predictors such as location, size, number of LNs involved, percentage of LNs involved, and extranodal extension, between Asian and the U.S. populations and among different countries. Additional international multicenter studies are necessary to assess the prognostic impact of LN characteristics in PTC.

The prognostic significance of LN involvement might differ according to the patient age at diagnosis. For patients with PTC aged 55 years or older, the data have consistently demonstrated decreased survival in the context of cervical LN metastases (38, 39). Our findings reported in the present study further corroborate that older patients ≥ 55 years presenting with N1 disease at presentation have poor PTCSS compared with patients with N0 disease. In younger patients with PTC, however, the overall impact of nodal metastases on long-term survival is debatable. Based on the current TNM staging system, patients < 55 years with PTC without distant metastases are all classified as having stage I disease even if they harbor lateral neck disease or extensive cervical LN metastases (9). However, recent large-scale cohort studies have reported N1b disease and an increasing number of positive nodes to be prognostic in patients less than 55 years of age at diagnosis of PTC (19, 35). In addition, large data examining the independent impact of LND on long-term PTCSS for patients less than 55 years are lacking.

Our study had several strengths. The SEER database provided a large volume of contemporary patients who were aged ≥ 55 years, which was associated with an increase in statistical power. In addition, the database contains cases from 18 registry areas in the United States, thereby helping to reduce the bias that could be derived from a single institutional study. Thus, the generalizability of the results was satisfactory. Owing to these notable strengths, the SEER registry data have been frequently utilized to study thyroid cancer staging (40–42). Our study also had some limitations. First, some selection biases were inevitable because of the retrospective and observational nature of this study. It was, therefore, difficult to draw an absolute conclusion that the TNnM staging system is superior to the 8th edition TNM classification for older patients with PTC. Second, our proposed staging system should be externally validated in a new cohort, although rigorous internal validation was conducted. Third, we were unable to control for potentially important variables that could have affected survival outcomes, such as vascular invasions, sizes of the largest positive LN, and extra-nodal extensions (43–45), owing to their absence from the data set. Finally, participants in this investigation were all aged ≥ 55 years and diagnosed with PTC, so further confirmation of the proposed TNnM staging system’s generalization into young populations and other thyroid cancer subtypes is needed.

## Conclusions

We established the critical importance of metastatic cervical LN burden in delineating the prognosis among PTC patients aged ≥ 55 years, with increased positive nodes conferring worse PTCSS. Moreover, we present a proposed TNnM staging scheme that incorporates metastatic node number into the current AJCC system to improve long-term PTCSS prediction. After external validation, the proposed system should be considered in future reviews of the AJCC 8th edition.

## Data availability statement

The original contributions presented in the study are included in the article/**Supplementary Material**. Further inquiries can be directed to the corresponding author.

## Author contributions

Study concepts and design: Y-GS. Data acquisition: FC, Q-LS. Quality control of data and algorithms: Y-GS, J-YT, X-CH. Data analysis and interpretation: Y-GS, X-CH. Statistical analysis: Y-GS, FC. Manuscript preparation, edition, and review: All authors. All authors contributed to the article and approved the submitted version.
