# Supplementary material for: The number of metastatic lymph nodes optimizes staging in patients aged 55 years or older with papillary thyroid cancer
Source: Front Endocrinol (Lausanne). 2022 Dec 9;13:1026737. doi: 10.3389/fendo.2022.1026737 (PMC9780272; doi:10.3389/fendo.2022.1026737)
Supplement: Supplementary file 1 [file Table_1.docx]

eTable

Possible combinations of 8th edition AJCC T/M category and Nn category and 10-year PTCSS for the training data set

|  | TNnM | No. (%) of Patients  (N = 4,971) | 10-year PTCSS, % |
| --- | --- | --- | --- |
| Combination 1 | T1N0nM0 | 2,221 (44.68) | 99.19 |
| Combination 2 | T2N0nM0 | 512 (10.30) | 97.28 |
| Combination 3 | T3N0nM0 | 156 (3.14) | 91.35 |
| Combination 4 | T4aN0nM0 | 84 (1.69) | 91.35 |
| Combination 5 | T4bN0nM0 | 37 (0.74) | 82.86 |
| Combination 6 | T1N1nM0 | 655 (13.18) | 97.56 |
| Combination 7 | T2N1nM0 | 292 (5.87) | 91.55 |
| Combination 8 | T3N1nM0 | 94 (1.89) | 85.25 |
| Combination 9 | T4aN1nM0 | 157 (3.16) | 76.74 |
| Combination 10 | T4bN1nM0 | 50 (1.01) | 74.69 |
| Combination 11 | T1N2nM0 | 159 (3.20) | 95.90 |
| Combination 12 | T2N2nM0 | 112 (2.25) | 89.77 |
| Combination 13 | T3N2nM0 | 58 (1.17) | 71.67 |
| Combination 14 | T4aN2nM0 | 80 (1.61) | 50.58 |
| Combination 15 | T4bN2nM0 | 27 (0.54) | 55.56 |
| Combination 16 | Any T, any Nn, M1 | 277 (5.57) | 58.52 |

Abbreviations: PTCSS, papillary thyroid cancer-specific survival; Nn, metastatic node number; N0n, no evidence of metastatic nodes; N1n, 1-4 metastatic nodes; N2n, > 4 metastatic nodes; AJCC, American Joint Committee on Cancer.
